# Bipolar mania with psychosis vs without psychosis: A clinical characterization with indirect measures of severity

**DOI:** 10.1192/j.eurpsy.2021.247

**Published:** 2021-08-13

**Authors:** F. Andrade, A.S. Machado, A. Vieira, A. Silva

**Affiliations:** Psychiatry Clinic, Centro Hospitalar e Universitário de São João, Porto, Portugal

**Keywords:** bipolar disorder, manía, psychotic simptoms, severity

## Abstract

**Introduction:**

The presence of psychotic symptoms is highest during acute episodes of bipolar mania. There is no evidence base regarding the implications of psychosis in the prognosis of bipolar disorder, despite common assumption that their occurrence reflects greater disease severity.

**Objectives:**

We aim to compare sociodemographic and clinical characteristics of inpatients admitted for bipolar mania with and without psychotic features.

**Methods:**

Retrospective observational study of inpatients admitted between January 1^st^ 2017 and 31 October 2020 in a psychiatry inpatient unit of a tertiary hospital. Descriptive analysis of the results was performed using the SPSS software, version 26.0.

**Results:**

Between 2017 and October 2020 there were 103 admissions due to mania bipolar I disorder, 53.4% (n=55) with psychotic symptoms. When compared with mania without psychosis, psychotic mania was associated to male gender (71.1% to 39.7%; c^2^(1, N = 103) = 10,06; p = 0.02) and younger age (t(103) = -2.43; p = 0.017). The proportion of compulsory admissions and average length of stay were similar between mania with psychosis and mania without psychosis. Also, having a manic bipolar episode with psychotic symptoms was not associated to being prescribed a long-acting injectable antipsychotic.

**Conclusions:**

The presence of psychotic symptoms in bipolar manic episodes were associated to male gender and younger age but not to indirect measures of illness severity.

**Disclosure:**

No significant relationships.

